# Effect of speed and leading or trailing limbs on surface muscle activities during canter in Thoroughbred horses

**DOI:** 10.1371/journal.pone.0286409

**Published:** 2023-05-26

**Authors:** Yuji Takahashi, Toshiyuki Takahashi, Kazutaka Mukai, Yusaku Ebisuda, Hajime Ohmura

**Affiliations:** Sports Science Division of Equine Research Institute, Japan Racing Association, Shimotsuke, Tochigi, Japan; Fondazione Policlinico Universitario Gemelli IRCCS, ITALY

## Abstract

Given that Thoroughbred horses’ canter is an asymmetric gait, not only speed but also leading or trailing limbs could affect muscle activities. However, the muscle activity during a canter remains poorly understood. Hence, we aimed to investigate speed and lead-side (leading or trailing) effects on surface electromyography (sEMG) during a canter. The sEMG data were recorded from left *Musculus brachiocephalicus* (Br), *M*. *infraspinatus* (Inf), long head of *M*. *triceps brachii* (TB), *M*. *gluteus medius* (GM), *M*. *semitendinosus* (ST), and *M*. *flexor digitorum longus* of seven Thoroughbreds with hoof-strain gauges at the left hooves. Horses cantered on a flat treadmill at 7, 10, and 13 m/s for 25 s each without lead change. Subsequently, the horses trotted for 3 min and cantered at the same speed and duration in the opposite lead side (“leading” at the left lead and “trailing” at the right lead). The order of the lead side and speed was randomized. The mean of 10 consecutive stride durations, duty factors, integrated-EMG values (iEMG) for a stride, and muscle onset and offset timing were compared using a generalized mixed model (*P* < 0.05). Stride durations and duty factors significantly decreased with speed regardless of the lead side. In all muscles, iEMG at 13 m/s significantly increased compared with 7 m/s (ranging from +15% to +134%). The lead-side effect was noted in the iEMG of Br (leading > trailing, +47%), Inf (leading > trailing, +19%), GM (leading < trailing, +20%), and ST (leading < trailing, +19%). In TB, GM, and ST, muscle onset in trailing was earlier than the leading, while offset in the leading was earlier in Br. In conclusion, different muscles have different responses to speed and lead side; thus, both the lead side and running speed should be considered during training and/or rehabilitation including canter or gallop.

## Introduction

In horses, locomotion is derived from a complex coordination of forces generated by muscle activation and elastic energy by tendons and ligaments. Understanding these mechanisms is essential for the equine industry to prevent injury or propose a training method. Until recently, several studies using surface electromyography (sEMG) have reported that the muscle activity during trotting increases with speed [1–5]. However, the effect of speed on muscle activity during canter and gallop [6], which are the gaits required in considerably high-intensity exercises (e.g., cross-country and horse racing), remains poorly known.

Canter and gallop are asymmetric gaits, whereas walking and trotting are symmetric gaits [7]. In symmetric gaits, ground reaction force and kinematics are similar in the left and right limbs of sound horses [7–9] but are different in asymmetric gaits [10, 11]. For example, the range of motion in elbow joints is higher in the leading limbs than in the trailing limbs, whereas that in hip joints is higher in the trailing limbs [10]. Furthermore, ground reaction force is higher in the trailing hind limb than in the leading limb at 10 m/s canter [11]. Thus, the leading and trailing limbs play different roles during a canter, and their muscle activities could differ according to each muscle’s function. By elucidating muscle activity differences between leading and trailing limbs, those involved with equine industry could make a better training and/or rehabilitation program.

This study aimed to investigate the effect of speed and lead side (leading or trailing) on muscle activity during a canter. We hypothesized that the muscle activity increases with speed and that some muscles exhibit a higher activity in the leading limbs and others in the trailing limbs.

## Materials and methods

### Ethics

The ethical and welfare regulations of the Animal Care Committee of the Equine Research Institute approved the study protocol (19–5).

### Horses

We used seven clinically healthy Thoroughbreds (5 geldings and 2 mares; median age: 4 years (range: 3–9), mean ± standard deviation of body weight: 502 ± 48 kg). All horses were familiar with running on a treadmill. Before the experiments, they were trained on a 6% inclined treadmill (SÄTO AB, Knivsta, Sweden) twice a week for 3 weeks, consisting of 3 min trot (3.5 m/s) followed by 7, 10, and 13 m/s canter for 30 s at both left and right leads. This protocol was performed on a 0% inclined treadmill in the last training before the experiment.

### Experimental study setup

The EMG signals of the *Musculus brachiocephalicus* (Br), *M*. *infraspinatus* (Inf), long head of *M*. *triceps brachii* (TB), *M*. *gluteus medius* (GM), *M*. *semitendinosus* (ST), and *M*. *flexor digitorum longus* (FDL) were recorded. In accordance with previous literature [1, 12–17], surface silver–silver chloride electrodes (H124SG, Covidien, MA, USA) with a diameter of 16 mm were attached to each muscle, parallel to the muscle fibers. We set a 25 mm distance between the center of each surface electrode, with active and reference electrodes set side by side. To ensure that the targeted muscle in each electrode location was not covered by other muscles, we verified it by ultrasonography as well as by the dissection of horses euthanized for other research projects.

The skin over each muscle and around the muscle belly were shaved and cleaned with alcohol. The electrodes were attached to the left-side muscles using a fast-acting glue (Gachi; Kokuyo Co., Osaka, Japan). The sEMG data were collected in the same way as our previous studies [14, 16, 17]. Briefly, the electrodes were connected to a compact electrode telemetry system—which had active, reference, and ground electrodes, including the wireless transmitter (ZB-150H, Nihon Kohden Corp., Tokyo, Japan)—via snap-type lead cables (TK-217-018, Unique Medical Co., Tokyo, Japan). The sEMG data from the transmitters attached by a foam pad (Foam pad 75A; Nihon Kohden Cop., Tokyo, Japan) on the horse’s body surface were stored and displayed in real time on a host computer (WEB-7000; Nihon Kohden Corp., Tokyo, Japan). If the noise was excessive or the electrodes were detached during measurement, the sEMG data were omitted from the analysis.

To detect foot-on and foot-off events, we firmly attached strain gauges (N22-FA-10-120-11-VS3; Showa Measuring Instruments, Inc., Tokyo, Japan) with glue (Gachi; Kokuyo Co., Osaka, Japan) to the dorsal midline of the left fore and hind hooves. Moreover, we synchronized the sEMG and strain gauge data by connecting dynamic strain-measuring instruments (DPM-612B; Kyowa Electronic Instruments Co., Tokyo, Japan) to the input terminal box (JC-130H; Nihon Kohden Corp., Tokyo, Japan) of the multichannel telemetry system by means of a coaxial cable with Bayonet Neill–Concelman connectors. Both the sEMG and hoof-strain gauge signals were collected at 1000 Hz with a 10 bit resolution (5 mV, full scale) and then filtered (band-pass filter including Bessel filter, 30–500 Hz for EMG; low-pass filter, 250 Hz for strain gauges).

### Experimental protocol

**Fig 1:**
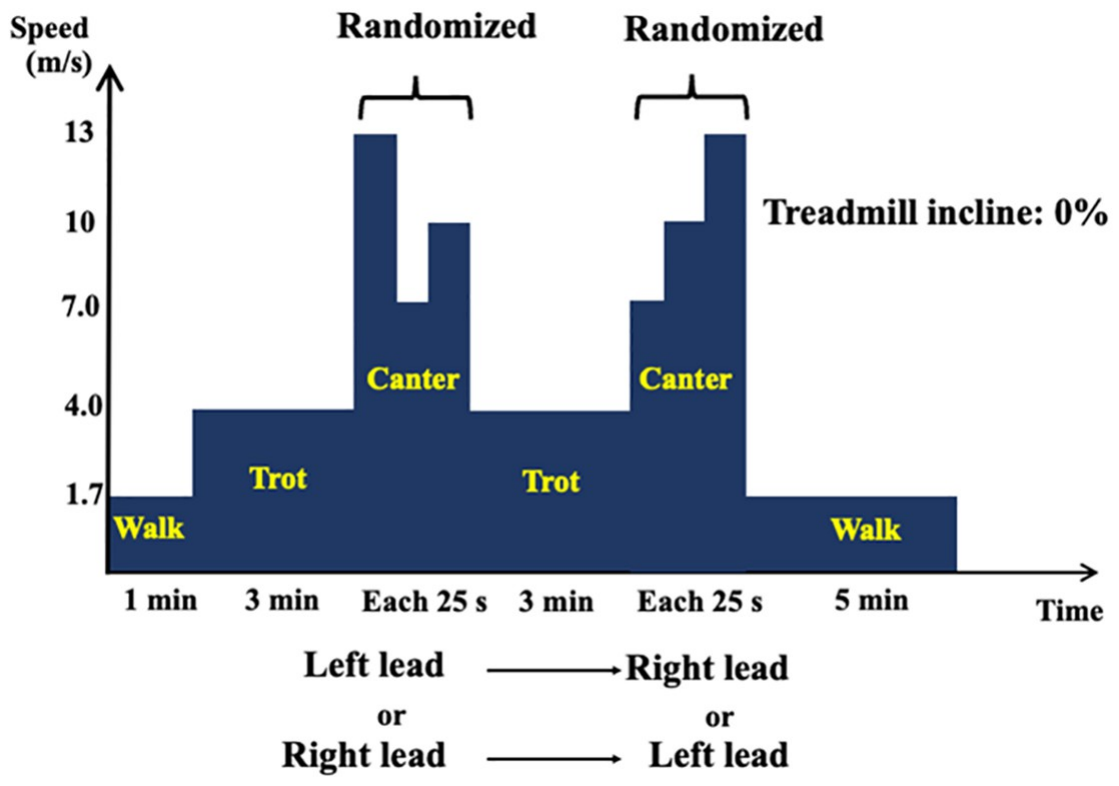
Experimental study design. After warming up by a 1 min walk and a 3 min trot, horses cantered at 7, 10, and 13 m/s for 25 s each randomly without a lead-limb change. Subsequently, they trotted for 3 min followed by the same canter protocol in the opposite lead side. Note that the measured muscle activity would be of “leading” when horses performed a left-lead canter, and “trailing” when horses performed a right-lead canter.

The experiment was conducted on the 0% inclined treadmill (SÄTO AB, Knivsta, Sweden). After warming up by a 1 min walk (1.7 m/s) and a 3 min trot (3.5 m/s), the horses cantered at 7, 10, and 13 m/s for 25 s each without a lead change (Fig 1). Subsequently, they trotted for 3 min and cantered again at the same speed and duration in the opposite lead side, which was considered as “leading” at the left lead and “trailing” at the right lead. The order of the lead side and speed was randomized. When the horses performed a lead-limb change during a canter, the speed decreased to make horses trot and canter again in the same lead side.

### Data analysis

We calculated the integrated-electromyography value (iEMG), which represents the area under the voltage curve of each muscle for a stride. The iEMG for 100 ms before the hoof strike, during stance time, and during the remaining swing phase were also calculated, followed by each phase’s ratio to iEMG for a stride. Furthermore, muscle activation onset and offset were detected by using enveloped signals and smoothed using a low-pass Butterworth 4^th^ order filter with 10 Hz cutoff frequency according to a recent publication [18] through a custom-made MATLAB (Release 2022b, The MathWorks Inc., Natick, Mass., USA) script. We defined the amplitude threshold as 10% of the peak amplitude of each individual sEMG signal, and the timing threshold as 5% of the average gait cycle duration across all horses [18]. Onset and offset events for each muscle were normalized to 0%–100% of stride cycle starting at the contact of each hoof.

For the statistical analysis, we used the mean of 10 consecutive strides. All statistical data were analyzed using a commercial software (SAS version 9.4; SAS Institute, Cary, NC, USA). To ascertain whether the effects of speed and lead side (leading or trailing) on stride parameter, iEMG for a stride, and muscle onset or offset timing were significant, we conducted a generalized linear mixed-effects model with gamma distribution and log link function (PROC GLIMMIX), with the speed and lead side as a fixed effect and the individual horse as a random effect. In addition, we employed Tukey’s multiple comparisons of least squared means to characterize the differences between categories. A *P* value of less than 0.05 was considered statistically significant.

## Results

Four horses were cantered in the right lead followed by the left lead, and three horses were cantered in the left lead followed by the right lead. Stride duration, stance time, and duty factor both in forelimb and hindlimb significantly decreased with speed (*P* < 0.001, Table 1), whereas the lead side did not affect any stride parameters (stride duration, *P* = 0.61; forelimb stance time, *P* = 0.28; forelimb duty factor, *P* = 0.35; hindlimb stance time, *P* = 0.30; hindlimb duty factor, *P* = 0.63). In post hoc test, significant changes were noted between each speed in each parameter (all *P* < 0.001, Table 1).

**Table 1:** Stride parameters at each speed both in leading and trailing limbs.

	7 m/s		10 m/s		13 m/s	
	Leading	Trailing	Leading	Trailing	Leading	Trailing
Stride duration (ms)	544 ± 13.5^a^	543 ± 17.2^a^	503 ± 10.4^b^	498 ± 9.60^b^	471 ± 5.63^c^	472 ± 6.54^c^
Forelimb stance time (ms)	168 ± 8.10^a^	169 ± 10.9^a^	131 ± 7.19^b^	129 ± 5.45^b^	110 ± 5.59^c^	106 ± 3.39^c^
Hindlimb stance time (ms)	172 ± 6.18^a^	168 ± 7.17^a^	135 ± 6.91^b^	134 ± 5.65^b^	111 ± 5.75^c^	112 ± 5.15^c^
Duty factor (forelimb)	0.309 ± 0.014^a^	0.312 ± 0.015^a^	0.262 ± 0.013^b^	0.258 ± 0.012^b^	0.233 ± 0.013^c^	0.225 ± 0.009^c^
Duty factor (hindlimb)	0.316 ± 0.011^a^	0.309 ± 0.014^a^	0.269 ± 0.013^b^	0.269 ± 0.014^b^	0.236 ± 0.013^c^	0.237 ± 0.013^c^

Data are expressed as mean ± standard deviation. The effects of speed were significant in all parameters, whereas those of lead side were not. Different letters in each row mean significant differences between speed and the corresponding parameter.

**Fig 2:**
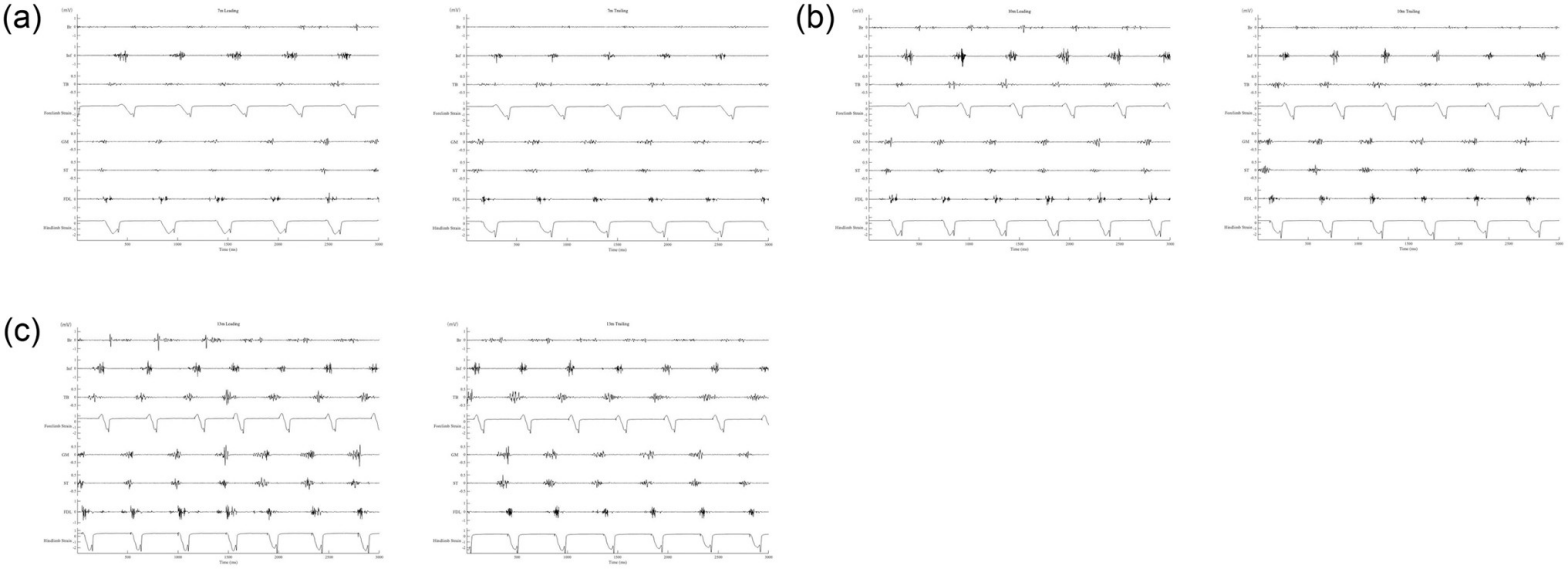
A representative electromyographic waveform in each muscle and strain gauge signals in the leading and trailing limbs. (a) 7, (b) 10, and (c) 13 m/s canters. Br; *Musculus brachiocephalicus*, Inf; *M*. *infraspinatus*, TB; long head of *M*. *triceps brachii*, GM; *M*. *gluteus medius*, ST; *M*. *semitendinosus*, FDL; *M*. *flexor digitorum longus*. Forelimb strain and hindlimb strain refer to signals from stain gauges attached to the hooves.

Fig 2 shows a representative of sEMG waveform of each muscle during the experiment. For statistical analysis, all data for Inf, GM, and ST were used (n = 7 at each speed both in the leading and trailing limbs). In FDL, we removed the data in one horse because the electrodes were detached during the warm-up (n = 6 at each speed both in the leading and trailing limbs). The data of 10 and 13 m/s TB in the leading limb and 13 m/s Br in the leading limb of one horse were also eliminated because of excessive noise, which makes the analysis difficult (Br: n = 6 at 13 m/s in the leading limb, n = 7 at other trials, TB; n = 6 at 10 m/s and 13 m/s in the leading limb, n = 7 at other trials).

**Fig 3:**
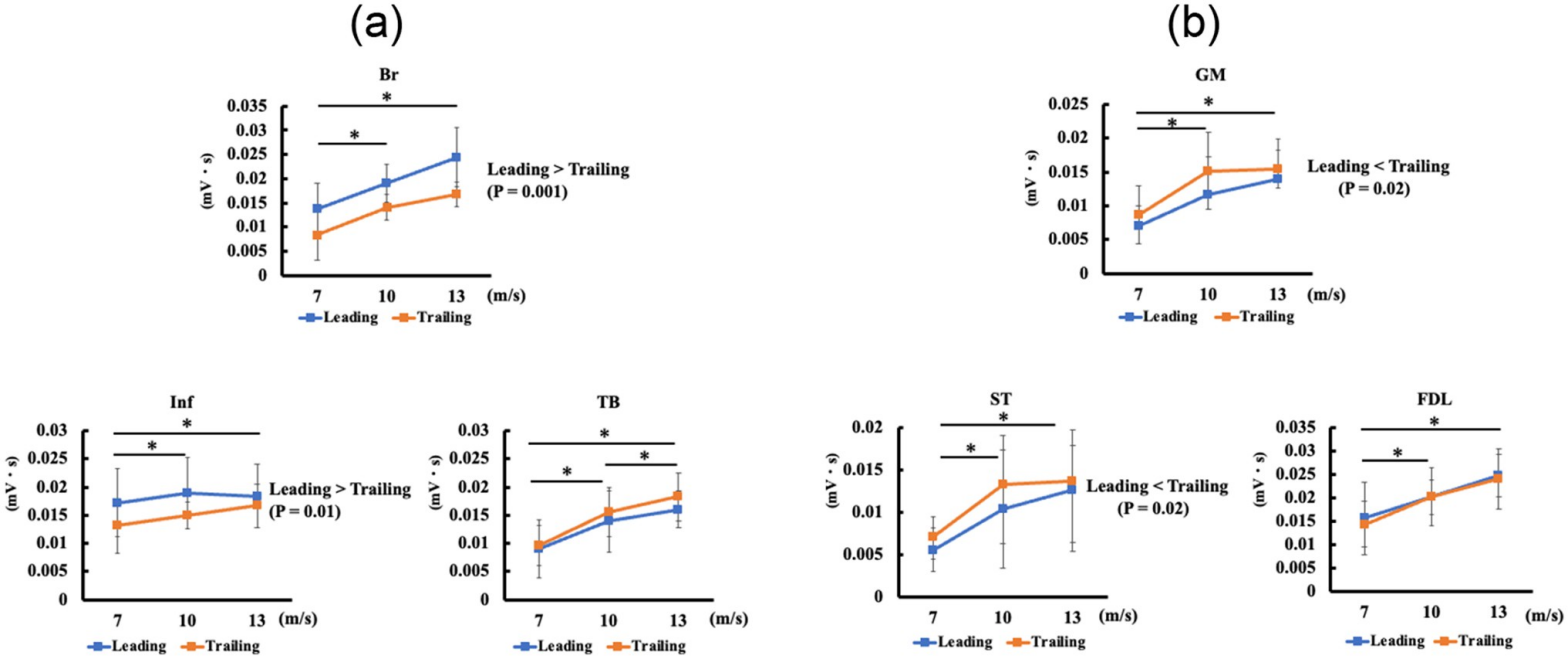
Effects of speed and lead side on each integrated-electromyography value (iEMG). (a) Forelimb muscle activity (Br; *Musculus brachiocephalicus*; Inf, *M*. *infraspinatus*; TB, long head of *M*. *triceps brachii*). (b) Hindlimb muscle activity (GM; *M*. *gluteus medius*, ST; *M*. *semitendinosus*, FDL; *M*. *flexor digitorum longus*). In all muscles, iEMG significantly increased with speed. The iEMG in Br and Inf was significantly higher in the leading than in the trailing. Conversely, the iEMG in GM and ST was significantly higher in the trailing than in the leading. Speed showed no interaction with the lead side. Data are shown as mean ± standard deviation. * indicates significant changes (*P* < 0.05) by generalized mixed model analysis followed by Tukey’s post hoc test.

There were no interactions between speed and lead side in any muscles. Fig 3 depicts the relationships of iEMG for a stride with speed and lead side. In all muscles, the iEMG for a stride significantly increased with speed (Inf, *P* = 0.01; other muscles, *P* < 0.001). Post hoc test showed significant differences between 7 and 13 m/s in all muscles (Br: +84%, *P* < 0.001; Inf: +15%, *P* = 0.02; TB: +134%, *P* < 0.001; GM: +86%, *P* < 0.001; ST: +109%, *P* < 0.001; FDL: +62%, *P* < 0.001). Additionally, all muscles (Br: +49%, Inf: +13%, TB: +90%, GM: +69%, ST: +88%, and FDL: +34%) showed significant increases between 7 and 10 m/s (Inf: *P* = 0.03, others: *P* < 0.001). Conversely, only TB showed significant differences between 10 and 13 m/s (Br, *P* = 0.11; Inf, *P* = 0.95; TB: +23%, *P* = 0.01; GM, *P* = 0.36; ST, *P* = 0.35; FDL, *P* = 0.05).

In Br and Inf, the iEMG for a stride of the leading was significantly higher than that of the trailing (Br: +47%, *P* = 0.001; Inf: +19%, *P* = 0.01; Fig 3a). In GM and ST, the trailing had a significantly higher iEMG than the leading (GM: +20%, *P* = 0.02; ST: +19%, *P* = 0.02; Fig 3b). In contrast, TB and FDL showed no significant lead-side effect (*P* = 0.10 and *P* = 0.65, respectively).

**Fig 4:**
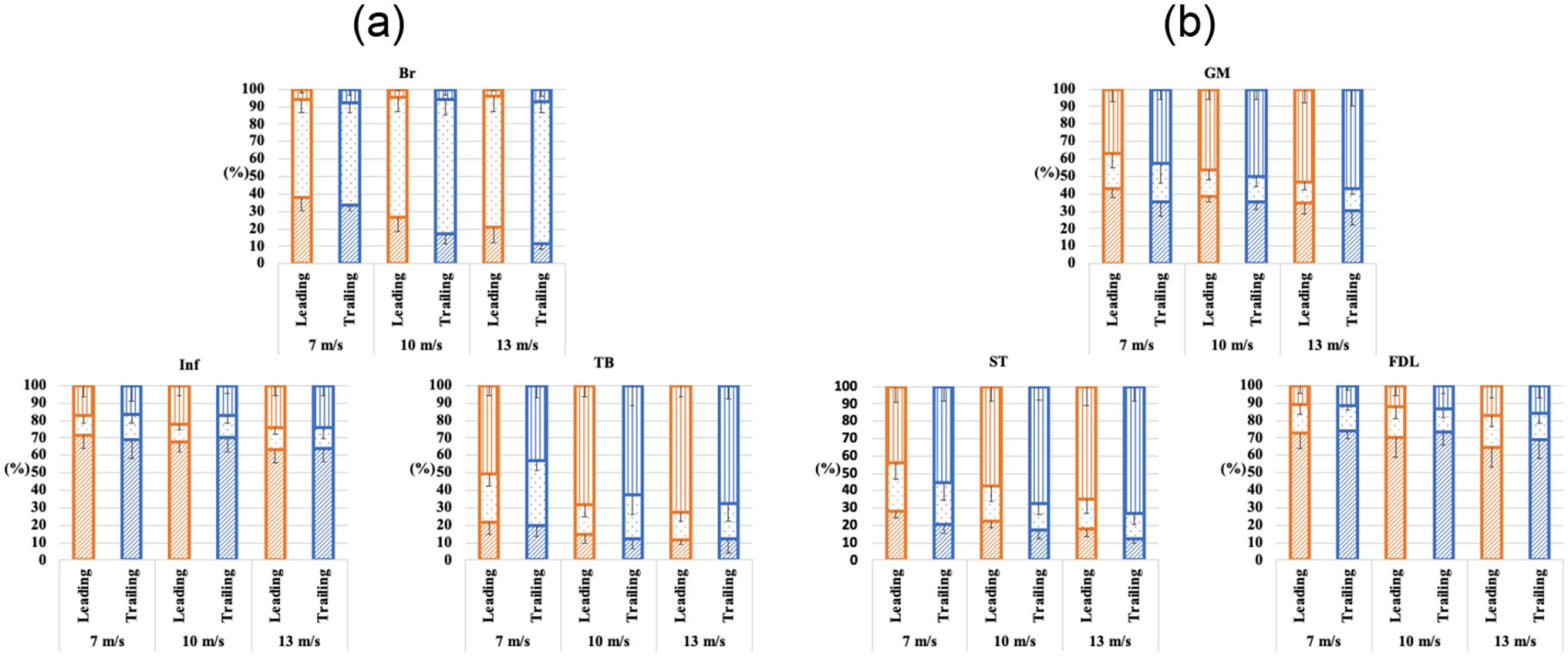
Stacked bar graphs with error bars (standard deviation) showing percentage of integrated-electromyography values (iEMG) during 100 ms before the hoof strike, the remaining swing phase, and stance phase. (a) Forelimb muscle activity (Br, *Musculus brachiocephalicus*; Inf, *M*. *infraspinatus*; TB, long head of *M*. *triceps brachii*). (b) Hindlimb muscle activity (GM, *M*. *gluteus medius*; ST, *M*. *semitendinosus*; FDL, *M*. *flexor digitorum longus*). Vertical, dotted, and diagonal lines indicate the 100 ms before the hoof strike, the remaining swing phase, and stance phase, respectively. As the speed increased, the percentage of iEMG during the stance phase decreased in Br, whereas that of iEMG for 100 ms before the hoof strike, increased in TB, GM, and ST. In Inf and FDL, slight increases in the percentage of iEMG for 100 ms before the hoof strike were found.

Fig 4 illustrates the proportion of iEMG during each phase to iEMG for a stride. The proportion of iEMG for 100 ms before the hoof strike increased with speed, especially in TB (leading, from 50% at 7 m/s to 73% at 13 m/s; trailing, from 43% at 7 m/s to 67% at 13 m/s), GM (leading, from 37% at 7 m/s to 53% at 13 m/s; trailing, from 43% at 7 m/s to 57% at 13 m/s), and ST (leading, from 44% at 7 m/s to 65% at 13 m/s; trailing, from 56% at 7 m/s to 73% at 13 m/s), whereas that in Inf (both leading and trailing, from 17% at 7 m/s to 24% at 13 m/s) and FDL (leading, from 11% at 7 m/s to 17% at 13 m/s; trailing, from 12% at 7 m/s to 16% at 13 m/s) showed slight increase. In Br, the proportion of iEMG during stance time decreased with speed, and it was considerably different between leading and trailing, especially in fast speed (leading, from 38% at 7 m/s to 21% at 13 m/s; trailing, from 33% at 7 m/s to 12% at 13 m/s). Mean values and standard deviations for each muscle at each speed are shown in the Supplementary information (S1 Table).

**Fig 5:**
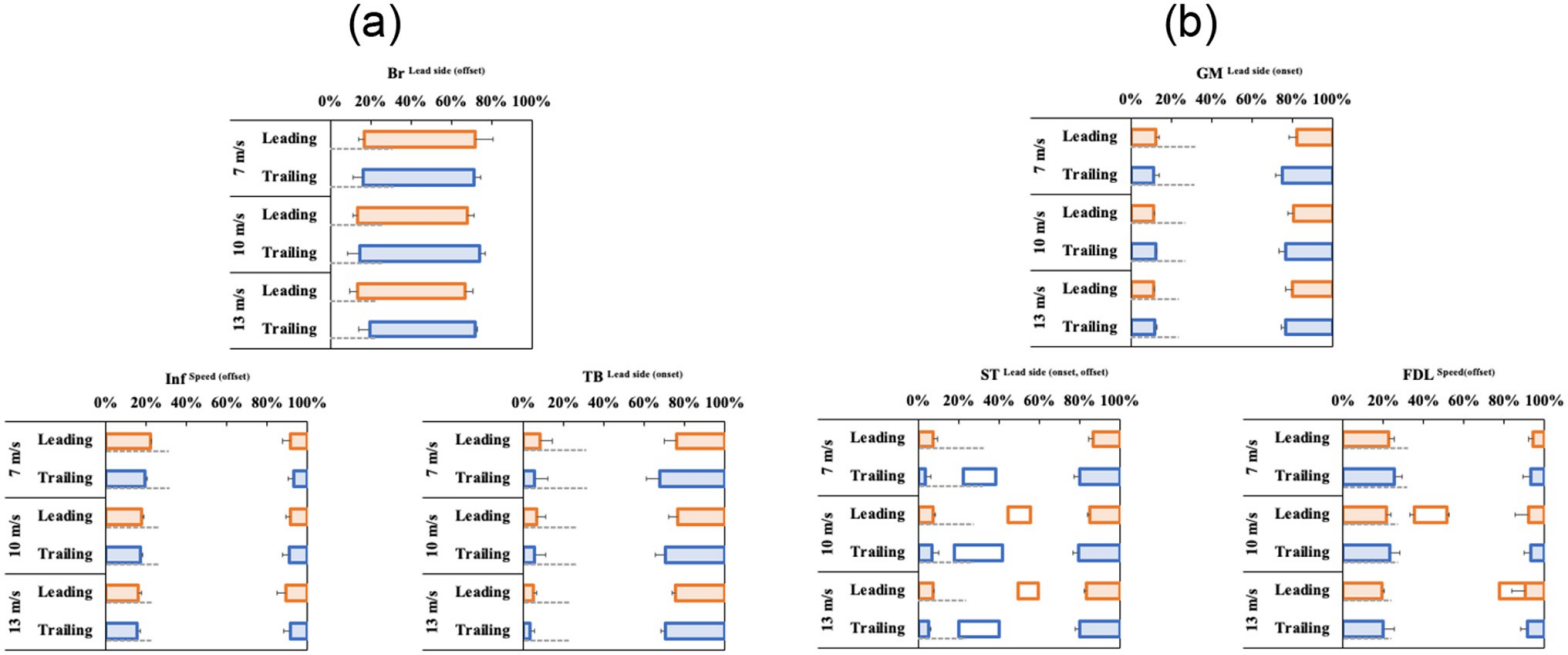
Periods of the electrical activity of forelimb muscles at each speed across a normalized stride cycle (0%–100% hoof strike to subsequent hoof strike). (a) Br, *Musculus brachiocephalicus*; Inf, *M*. *infraspinatus*; TB, long head of *M*. *triceps brachii*. (b) GM; *M*, *gluteus medius*, ST; *M*. *semitendinosus*, FDL; *M*. *flexor digitorum longus*). The Br is activated at the middle stance phase and deactivated at the middle swing phase; other muscles are activated before the hoof contact and deactivated during the stance phases. Error bars represent one standard deviation of the mean onset and offset. The small characters (speed or lead sides) at the right shoulder of each muscle name indicate the significant effects (*P* < 0.05) of each fixed effect by generalized linear mixed model analysis in terms of muscle onset or offset timing described in the parentheses. Some horses had additional muscle activation (shown in white boxes, 1 horse in ST and FDL at 13 m/s in the leading, 2 horses in FDL at 10 m/s in the leading). The gray dashed horizontal lines represent the stance phase and the specified values in each limb at each speed are described as duty factor in Table 1.

Fig 5 shows the onset and offset timing of each muscle across the gait cycle as a percentage of time. The Br was activated between the middle stance phase and middle swing phase. The other muscles, except Br, became active during the late swing phase and remained active until the early (in TB, and ST), the middle (in GM), or the late stance phases (in Inf and FDL). In addition, one horse showed ST muscle activation from the late stance to the early swing phase in the trailing limb and from the early to middle swing phase in the leading limb, and two horses displayed FDL muscle activation in the early swing phase in the leading limb at 10 m/s. Mean values and standard deviations of muscle onset and offset timing of each muscle at each speed are shown in the Supplementary information (S2 Table).

The speed did not affect the onset timing of any muscles (Br, *P* = 0.32; Inf, *P* = 0.09; TB, *P* = 0.16; GM, *P* = 0.87; ST, *P* = 0.15; FDL, *P* = 0.08). It also did not affect the offset timing of Br (*P* = 0.33), TB (*P* = 0.06), GM (*P* = 0.74), and ST (*P* = 0.13), but as it increased, Inf (*P* < 0.001) and FDL (*P* = 0.02) showed an earlier offset timing. Moreover, significant differences were observed between the speeds in Inf (7 m/s vs. 10 m/s, *P* < 0.001; 7 m/s vs. 13 m/s, *P* < 0.001; 10 m/s vs. 13 m/s, *P* = 0.009), but in FDL, the significant difference was found only between 7 and 13 m/s (*P* = 0.02) (7 m/s vs. 10 m/s, *P* = 0.45; 10 m/s vs. 13 m/s; *P* = 0.14). In some muscles, the lead side affected the muscle onset and offset timing. For instance, the offset timing of the trailing in Br was significantly delayed compared with the leading (*P* = 0.04), while the onset timing was not affected by the lead side (*P* = 0.19). In Inf, neither the onset timing (*P* = 0.05) nor offset timing (*P* = 0.06) demonstrated significant differences. As for TB, GM, and ST, the onset timing of the trailing was significantly earlier than the leading (all: *P* < 0.001). In contrast, the offset timing of the leading in ST was significantly delayed compared with the trailing (*P* = 0.02), whereas no effects were found in TB (*P* = 0.11) and GM (*P* = 0.84). In FDL, the lead side did not affect muscle onset (*P* = 0.71) or muscle offset (*P* = 0.19) timing.

## Discussion

The present study demonstrated that the muscle activity increased as the speed increased during a canter, which is an asymmetric gait. This result is similar to that observed during trotting, which is a symmetric gait [1–5]. Physiologically, speed increase requires muscle recruitments, leading to large oxygen uptake [19]. In Thoroughbreds, oxygen uptake at 13 m/s was almost three times higher than that at 7 m/s on a flat treadmill [19]. In the present study, iEMG increase ranged from 15% to 134% in each muscle at 13 m/s in comparison with 7 m/s. These small increases relative to oxygen uptake could be attributed to the sEMG property, that is, the electrodes are small and superficial; nonetheless, the muscle volume is large, relative to the region sampled by the electrodes, as discussed by Wickler et al. [20]. Interestingly, the increase rate differed in each muscle. For example, compared to the iEMG at 7 m/s, iEMG in the Inf at 13 m/s increased only by 15%, whereas that in TB and ST at 13 m/s increased by more than 100%. Thus, the muscle recruitment pattern against speed may depend on many properties, such as muscle function or muscle fiber length and fiber type composition. Further study is needed to elucidate the relationship between muscle activity and speed.

Our results showed that muscle activities in each phase during one-stride cycle could differ according to muscle functions and speed. In TB, GM, and ST, the speed of horses during canter was directly related to the proportion of muscle activities needed during precontact. It is likely that muscle activities during precontact could be essential at high-speed canter, which would be similar to human sprint running [21, 22] or trotting in horses [1]. Although these changes could be related to changes in stride parameters, the changes observed in TB, GM, and ST were considerably greater than those of stride parameters because the ratio of 100 ms to stride duration changed by only 3% between 7 and 13 m/s. Therefore, the increase of proportion in this phase could be presumably caused by the increase of muscle activity itself before the contact. Both in humans and horses, speed increase requires large ground reaction forces and short stance time [11, 23]. The precontact muscle activity could increase muscle stiffness, which is needed to resist high impacts at the very first stages of contact [21, 22] or required for increasing stride frequency and speed [24, 25]. Further, these muscles could be associated with limb deceleration and redirection from protraction to retraction at the late swing phase [26, 27]. These roles might become more important as horses run faster, considering the faster movement of the limbs. Conversely, the Inf and FDL, known as antigravity muscles that resist ground reaction force during the stance phase [28, 29], may not have such deceleration or redirection roles as TB, GM, and ST.

The muscle activity ratio in Br during the stance phase decreased with speed in both lead sides. This result could be associated with approximately 8% decrease of duty factor at 13 m/s compared with 7 m/s. Still, the muscle activity ratio during the stance phase to one stride considerably decreased, especially in the trailing limb, suggesting that the muscle activity during the swing phase became increasingly important as the horses ran faster. This result could be reasonable because more forelimb protraction is needed as the speed increases [30]. However, head and neck movement should also be considered because it is associated with the Br muscle activity [31]. The relationship between head and neck movements and muscle activity in Br during canter needs to be further investigated.

Although all muscles showed an increase in iEMG with speed, a statistically significant increase between 10 and 13 m/s was found only in TB, which was a relatively small increase (+23%). Oxygen uptake on a flat treadmill at 13 m/s increased by approximately 50% compared with that at 10 m/s, while that at 10 m/s was higher by 100% than at 7 m/s [19]. Possibly, sEMG measurement in a small and superficial area could not detect muscle recruitment changes between 10 and 13 m/s. Additionally, the percentage of iEMG for 100 ms before the hoof strike increased considerably, especially in TB, GM, and ST. Thus, the iEMG derived from the increase of eccentric-type contraction essential for increasing muscle stiffness might partially explain about small increase in iEMG in TB relative to oxygen uptake increase and no differences in iEMG in GM and ST between 10 m/s and 13 m/s because iEMG during eccentric contraction was less than the concentric-type contraction because of less fiber recruitments to exert any given forces [32, 33].

Upon comparing muscle activities between the leading side and trailing sides, muscle activities in the leading limb were found to be higher than those in the trailing limb in the Br and Inf, whereas muscle activities in the trailing limb were higher than those in the leading limb in the GM and ST. Given that the stride duration did not change according to the lead side, more muscle fiber recruitments were most likely required in the leading limbs in Br and Inf and in the trailing limbs in GM and ST. From these results, when horses run in one lead side, physical or morphological aspects such as different recruitment patterns or cross-sectional area exhibit more asymmetry between the left and right muscles. In addition to different kinematics between the leading and trailing limbs [10], varied muscle onset and offset timing could affect muscle contraction type (concentric or eccentric) or length during muscle discharge, possibly associated with force–velocity and force–length relationships [34, 35]. Further research that is synchronized with other equipment measuring 3D kinematics leading to inverse dynamics or muscle fiber length such as sonomicrometry, is needed.

The current study has some limitations. First, we could not statistically analyze our horse’s lead-limb preferences. Although our horses were trained to run in both lead sides, some horses had lead-limb preferences when galloping [36, 37]. Second, our experimental design was not a complete randomized protocol because running in the nonpreferred leading limb for several times would be difficult for the horses and such trials could detach sEMG electrodes. Third, we might have missed some activation because the signal processing we applied for detecting muscle onset and offset was developed from *M*. *biceps femoris* and *M*. *triceps brachii* [18]. Occasionally, the ST and FDL showed bimodal waveforms [12, 13], and the small discharge might have been missed if the peak values were considerably high. Finally, we must consider the differences between the treadmill and other types of surfaces, as these differences can affect the biomechanical parameter. In horses, exercises performed on the treadmill increased the fetlock joint range of motion and stride length as compared to the exercises performed on the overground surface [38, 39]. Harrison et al. detected insignificant differences in the muscle amplitude or timing of muscle activation while walking and trotting on the treadmill and overground surfaces for the majority of the studied muscles [40]; however, no study has compared the muscle activities while exercising on the treadmill and overground surfaces during canter. Investigating muscle activity differences on different surface types including the treadmill would be helpful for the equine industry.

## Conclusions

When horses cantered, different muscles showed different responses to speed and lead side. Especially in TB, GM, and ST, which are located proximally and considered to have a propulsive function, the muscle activity during the precontact phase increased as the speed increased. This finding might be associated with the increased stiffness of the muscle or leg to increase stride frequency. Further, some muscles had higher activities in the leading limb, while others in the trailing, suggesting that canter in both lead sides should be conducted during training or rehabilitation to avoid physiological and morphological asymmetry when cantering or galloping.

## Supporting information

S1 Table Descriptive statistic (mean ± standard deviation) for percentage of percentage of integrated-electromyography values (iEMG) during 100 ms before the hoof strike, the remaining swing phase, and stance phase. (XLSX)

S2 Table Descriptive statistics (mean ± standard deviation) for muscle onset and offset timing. (XLSX)

## References

[1] Robert C, Valette JP, Denoix JM. The effects of treadmill inclination and speed on the activity of two hindlimb muscles in the trotting horse. Equine Vet J. 2000;32: 312–317. doi: 10.2746/042516400777032246 10952380

[2] Robert C, Valette JP, Pourcelot P, Audigié F, Denoix JM. Effects of trotting speed on muscle activity and kinematics in saddlehorses. Equine Vet J. 2002;34: 295–301. doi: 10.1111/j.2042-3306.2002.tb05436.x 12405704

[3] Crook TC, Wilson A, Hodson-Tole E. The effect of treadmill speed and gradient on equine hindlimb muscle activity. Equine Vet J. 2010;42: 412–416. 21059038 10.1111/j.2042-3306.2010.00222.x

[4] Robert C, Valette JP, Denoix JM. The effects of treadmill inclination and speed on the activity of three trunk muscles in the trotting horse. Equine Vet J. 2001;33: 466–472. doi: 10.2746/042516401776254745 11558741

[5] Hoyt DF, Wickler SJ, Biewener AA, Cogger EA, De La Paz KL. In vivo muscle function vs speed. I. Muscle strain in relation to length change of the muscle-tendon unit. J Exp Biol. 2005;208: 1175–1190. doi: 10.1242/jeb.01486 15767316

[6] Busse NI, Gonzalez ML, Krason ML, Johnson SE. β-Hydroxy β-methylbutyrate supplementation to adult Thoroughbred geldings increases type IIA fiber content in the gluteus medius. J Anim Sci. 2021;99. 10.1093/jas/skab264 PMC8493890 34516615

[7] Barrey E. Gaits and interlimb coordination. In: Back W, Clayton HM, editors. Equine locomotion. 2nd ed. London: Saunders; 2013. pp. 85–97.

[8] Merkens HW, Schamhardt HC, Hartman W, Kersjes AW. Ground reaction force patterns of Dutch Warmblood horses at normal walk. Equine Vet J. 1986;18: 207–214. doi: 10.1111/j.2042-3306.1986.tb03600.x 3732241

[9] Merkens HW, Schamhardt HC, Van Osch GJ, Van Den Bogert AJ. Ground reaction force patterns of Dutch Warmblood horses at normal trot. Equine Vet J. 1993;25: 134–137. doi: 10.1111/j.2042-3306.1993.tb02923.x 8467772

[10] Back W, Schamhardt HC, Barneveld A. Kinematic comparison of the leading and trailing fore-and hindlimbs at the canter. Equine Vet J. 1997;29: 80–83. doi: 10.1111/j.2042-3306.1997.tb05060.x 9354296

[11] Self Davies ZT, Spence AJ, Wilson AM. Ground reaction forces of overground galloping in ridden Thoroughbred racehorses. J Exp Biol. 2019;222: jeb204107. doi: 10.1242/jeb.204107 31444280

[12] Jansen MO, Van Raaij JAGM, Van Den Bogert AJ, Schamhardt HC, Hartman W. Quantitative analysis of computer-averaged electromyographic profiles of intrinsic limb muscles in ponies at the walk. Am J Vet Res. 1992;53: 2343–2349. 1476320

[13] Robert C, Valette JP, Degueurce C, Denoix JM. Correlation between surface electromyography and kinematics of the hindlimb of horses at trot on a treadmill. Cells Tissues Organs. 1999;165: 113–122. doi: 10.1159/000016681 10516424

[14] Takahashi Y, Mukai K, Matsui A, Ohmura H, Takahashi T. Electromyographic changes in hind limbs of Thoroughbreds with fatigue induced by treadmill exercise. Am J Vet Res. 2018;79: 828–835. doi: 10.2460/ajvr.79.8.828 30058845

[15] St George L, Hobbs SJ, Richards J, Sinclair J, Holt D, Roy SH. The effect of cut-off frequency when high-pass filtering equine sEMG signals during locomotion. J Electromyogr Kinesiol. 2018;43: 28–40. doi: 10.1016/j.jelekin.2018.09.001 30219734

[16] Takahashi Y, Mukai K, Ohmura H, Takahashi T. Do muscle activities of M. splenius and M. brachiocepalicus decrease due to exercise-induced fatigue in Thoroughbred horses? J Equine Vet Sci. 2020;86: 102901. 32067667 10.1016/j.jevs.2019.102901

[17] Takahashi Y, Mukai K, Ohmura H, Takahashi T. Changes in muscle activity with exercise-induced fatigue in Thoroughbred horses. Comp Exerc Physiol. 2021;17: 25–34.

[18] St George L, Clayton HM, Sinclair J, Richards J, Roy SH, Hobbs SJ. Muscle function and kinematics during submaximal equine jumping: What can objective outcomes tell us about athletic performance indicators? Animals (Basel). 2021;11. doi: 10.3390/ani11020414 33562875 PMC7915507

[19] Eaton MD, Evans DL, Hodgson DR, Rose RJ. Effect of treadmill incline and speed on metabolic rate during exercise in thoroughbred horses. J Appl Physiol (1985). 1995;79: 951–957. doi: 10.1152/jappl.1995.79.3.951 8567539

[20] Wickler SJ, Hoyt DF, Biewener AA, Cogger EA, De La Paz KL. In vivo muscle function vs speed. II. Muscle function trotting up an incline. J Exp Biol. 2005;208: 1191–1200. doi: 10.1242/jeb.01485 15767317

[21] Mero A, Komi PV. Electromyographic activity in sprinting at speeds ranging from sub-maximal to supra-maximal. Med Sci Sports Exerc. 1987;19: 266–274. 3600241

[22] Kyröläinen H, Avela J, Komi PV. Changes in muscle activity with increasing running speed. J Sports Sci. 2005;23: 1101–1109. doi: 10.1080/02640410400021575 16194986

[23] Weyand PG, Sternlight DB, Bellizzi MJ, Wright S. Faster top running speeds are achieved with greater ground forces not more rapid leg movements. J Appl Physiol (1985). 2000;89: 1991–1999. doi: 10.1152/jappl.2000.89.5.1991 11053354

[24] Farley CT, Glasheen J, McMahon TA. Running springs: speed and animal size. J Exp Biol. 1993;185: 71–86. doi: 10.1242/jeb.185.1.71 8294853

[25] Farley CT, González O. Leg stiffness and stride frequency in human running. J Biomech. 1996;29: 181–186. doi: 10.1016/0021-9290(95)00029-1 8849811

[26] Watson JC, Wilson AM. Muscle architecture of biceps brachii, triceps brachii and supraspinatus in the horse. J Anat. 2007;210: 32–40. doi: 10.1111/j.1469-7580.2006.00669.x 17229281 PMC2100266

[27] Deban SM, Schilling N, Carrier DR. Activity of extrinsic limb muscles in dogs at walk, trot and gallop. J Exp Biol. 2012;215: 287–300. doi: 10.1242/jeb.063230 22189773

[28] Clayton HM, Chateau H, Back W. Forelimb function. In: Back W, Clayton HM, editors. Equine locomotion. 2nd ed. London: Saunders; 2013. pp. 99–125.

[29] Clayton HM, Back W. Hindlimb function. In: Back W, Clayton HM, editors. Equine locomotion. 2nd ed. London: Saunders; 2013. pp. 127–145.

[30] Van Weeren PR, Van Den Bogert AJ, Back W, Bruin G, Barneveld A. Kinematics of the standardbred trotter measured at 6, 7, 8 and 9 m/s on a treadmill, before and after 5 months of prerace training. Acta Anat (Basel). 1993;146: 154–161. doi: 10.1159/000147438 8470459

[31] Kienapfel K. The effect of three different head-neck positions on the average EMG activity of three important neck muscles in the horse. J Anim Physiol Anim Nutr (Berl). 2015;99: 132–138. doi: 10.1111/jpn.12210 24954642

[32] Abbott BC, Aubert XM. The force exerted by active striated muscle during and after change of length. J Physiol. 1952;117: 77–86. 14946730 PMC1392571

[33] Bigland-Ritchie B, Woods JJ. Integrated electromyogram and oxygen uptake during positive and negative work. J Physiol. 1976;260: 267–277. doi: 10.1113/jphysiol.1976.sp011515 978517 PMC1309091

[34] Gordon AM, Huxley AF, Julian FJ. The variation in isometric tension with sarcomere length in vertebrate muscle fibres. J Physiol. 1966;184: 170–192. doi: 10.1113/jphysiol.1966.sp007909 5921536 PMC1357553

[35] Hill AV. The heat of shortening and the dynamic constants of muscle. Proc R Soc Lond B Biol Sci. 1938;126: 136–195. 10.1098/rspb.1949.0019 18152150

[36] Williams DE, Norris BJ. Laterality in stride pattern preferences in racehorses. Anim Behav. 2007;74: 941–950.

[37] Cully P, Nielsen B, Lancaster B, Martin J, McGreevy P. The laterality of the gallop gait in Thoroughbred racehorses. PLOS ONE. 2018;13: e0198545. doi: 10.1371/journal.pone.0198545 29883459 PMC5993273

[38] Barrey E, Galloux P, Valette JP, Auvinet B, Wolter R. Stride characteristics of overground versus treadmill locomotion in the saddle horse. Acta Anat. 1993;146: 90–94. doi: 10.1159/000147427 8470471

[39] Mendez-Angulo JL, Firshman AM, Groschen DM, Kieffer PJ, Trumble TN. Impact of walking surface on the range of motion of equine distal limb joints for rehabilitation purposes. Vet J. 2014;199: 413–418. doi: 10.1016/j.tvjl.2013.12.001 24556081

[40] Harrison SM, Whitton RC, King M, Haussler KK, Kawcak CE, Stover SM, Pandy MG. Forelimb muscle activity during equine locomotion. J Exp Biol. 2012;215: 2980–2991. doi: 10.1242/jeb.065441 22875767

